# HER2 as a novel therapeutic target for cervical cancer

**DOI:** 10.18632/oncotarget.5283

**Published:** 2015-09-21

**Authors:** Doo-Yi Oh, Seokhwi Kim, Yoon-La Choi, Young Jae Cho, Ensel Oh, Jung-Joo Choi, Kyungsoo Jung, Ji-Young Song, Suzie E. Ahn, Byoung-Gie Kim, Duk-Soo Bae, Woong-Yang Park, Jeong-Won Lee, Sangyong Song

**Affiliations:** ^1^ Department of Pathology and Translational Genomics, Samsung Medical Center, Sungkyunkwan University School of Medicine, Seoul, Korea; ^2^ Institute for Refractory Cancer Research, Samsung Medical Center, Seoul, Korea; ^3^ Department of Health Sciences and Technology, SAIHST, Sungkyunkwan University, Seoul, Korea; ^4^ Department of Obstetrics and Gynecology, Samsung Medical Center, Sungkyunkwan University School of Medicine, Seoul, Korea; ^5^ Samsung Genome Institute, Samsung Medical Center, Seoul, Korea

**Keywords:** HER2, cervical cancer, targeted therapy, patient-derived xenograft, trastuzumab

## Abstract

Surgery and radiation are the current standard treatments for cervical cancer. However, there is no effective therapy for metastatic or recurrent cases, necessitating the identification of therapeutic targets. In order to create preclinical models for screening potential therapeutic targets, we established 14 patient-derived xenograft (PDX) models of cervical cancers using subrenal implantation methods. Serially passaged PDX tumors retained the histopathologic and genomic features of the original tumors. Among the 9 molecularly profiled cervical cancer patient samples, a *HER2*-amplified tumor was detected by array comparative genomic hybridization and targeted next-generation sequencing. We confirmed HER2 overexpression in the tumor and serially passaged PDX. Co-administration of trastuzumab and lapatinib in the HER2-overexpressed PDX significantly inhibited tumor growth compared to the control. Thus, we established histopathologically and genomically homologous PDX models of cervical cancer using subrenal implantation. Furthermore, we propose HER2 inhibitor-based therapy for *HER2*-amplified cervical cancer refractory to conventional therapy.

## INTRODUCTION

Cervical cancer is the third most common cancer and fourth leading cause of death in women worldwide [1]. Radiotherapy and cisplatin-based chemo-radiation in addition to surgery are performed for curative treatment according to the clinical stage of the tumor and surgical findings [2]. However, patients with advanced or recurrent disease have a poor prognosis despite using of cisplatin-based combination chemotherapy [3]. Therefore, there is an increasing need for novel therapies that can replace or be added to the current therapy for these patients. Several targeted molecules including anti-vascular endothelial growth factor (VEGF) inhibitors, anti-epidermal growth factor receptor (EGFR) antibodies, and mammalian target of rapamycin (mTOR) inhibitors have been tested for recurrent cervical cancers, showing some promising results for certain patient subsets [4].

Xenografts derived from established cancer cell lines have been established but lack diverse histologic and molecular characteristics and tend to be derived from more aggressive tumors [5]. Unlike these cell line xenografts from cell lines, which cannot represent the complex tumor heterogeneity, xenografts using human cancer tissues mostly resolve these problems and adequately reflect the molecular features of patient tumors [6]. Patient-derived xenografts (PDXs) have been used for decades to screen for candidate drugs and monitor treatment response. Different models that vary according to tumor origin and implantation site are available. Several PDXs have been established for various cancers including colorectal cancer, bladder cancer, and glioblastoma [7–9]. However, cervical cancer PDXs have not so widely studied despite their potential benefits for drug testing and treatment response monitoring except one recent report using orthotopic model [10].

HER2 gene amplification or protein expression can be identified in various cancers other than breast; its incidence ranges from 1% to 12% in cervical cancers [11, 12]. HER2 expression in cervical cancer has been reported to be present in recurrent tumors [13] and that its expression is related to poor prognosis in locally advanced cervical cancer [14]. Recent whole-exome sequencing studies provided evidence of *HER2* activation by somatic mutation, amplification, and human papilloma virus (HPV) integration in cervical cancer [12], warranting investigation of the effects of HER2 inhibitors.

In this study, we tried to establish PDX using human cervical cancer tissues and find the new therapeutic strategy using this model. Here, we established PDXs derived from cervical cancer patients using subrenal capsule implantation models. In addition, when analyzing patient's tumors and PDXs, we found a single case with aberrant HER2 amplification and expression. Subsequent histopathologic and genomic characterization of the tumors in addition to monitoring response to anti-HER2 therapy in PDXs was performed. These results suggest that HER2 inhibitors-based therapy can be considered to on a platform that accurately mimics cervical cancer patients.

## RESULTS

### Establishment of cervical cancer PDXs

#### Clinicopathological characteristics

Of the 21 patient samples implanted, 14 were successfully engrafted into mice to create PDX models (engraftment rate, 66.7%; Table 1). The patients' ages ranged from 27 to 67 years (median, 49 years). Tumor samples were taken from the cervix of the radical hysterectomy specimens during surgery, except in 1 case (CX13) in which the sample was taken from lymph node tissue after excision because of recurrence in the inguinal lymph nodes. The HPV genotyping showed 19 samples were positive for HPV, all of which were high-risk types. Local recurrence was identified in 4 patients during routine follow-up. We did not find the difference of success rate according to histologic subtypes: squamous cell carcinoma (12/17, 70.6%) vs. adenocarcinoma (2/4, 50%).

**Table 1:** Clinicopathological characteristics of the cervical cancer patients and development of PDXs

No	ID	Age	Tumor location	Pathology	FiGO stage	LN status	Tumor size (cm)	Parametrial invasion	Vaginal RM	HPV	Surgery	Adjuvant therapy	Recurrence	DFS (Mo)	PDX success (Time from primary tumor to M1 [month])
1	CX1	54	cervix	SCC	IB2	Positive	4.5	Negative	Negative	NA	RH + LND	CCRT	No	32	No
2	CX3	44	cervix	SCC	IIB	NA	6	NA	NA	NA	Not done	CCRT	No	36	No
3	CX4	41	cervix	SCC	IB2	Negative	7	Negative	Negative	16	RH + LND	CCRT	No	34	Yes, 3mo
4	CX6	46	cervix	SCC	IB1	Negative	2.1	Negative	Negative	33	RH + LND	RT	No	32	Yes, 3mo
5	CX5	38	cervix	AC	IB2	Negative	10	Negative	Negative	16	RH + LND	not done	No	31	No
6	CX7	35	cervix	SCC	IB1	Positive	3.5	Negative	Negative	16	RH + LND	CCRT	No	31	Yes, 4mo
7	CX8	66	cervix	SCC	IIB	Positive	6	Negative	Negative	16	RH + LND	CCRT	No	24	Yes, 3mo
8	CX9	62	cervix	AC	IIB	Negative	3.6	Positive	Positive	16	RH + LND	CCRT	No	24	No
9	CX10	65	cervix	SCC	IB1	Negative	5.4	Negative	Negative	31	RH + LND	RT	No	27	Yes, 10mo
10	CX11	30	cervix	SCC	IIA	Positive	10	Positive	Negative	16,18	RH + LND	CCRT	No	29	Yes, 10mo
11	CX12	58	cervix	SCC	IB1	Positive	3.5	Negative	Negative	58	RH + LND	CCRT	No	26	No
12	CX13	55	lymph node	SCC	IIA	Positive	4	Positive	Negative	18	RH + LND	CCRT	Yes	13	Yes, 7mo
13	CX14	56	cervix	AC	IIB	Negative	4.5	Positive	Negative	31	RH + LND	not done	Yes	12	Yes, 3mo
14	CX15	67	cervix	SCC	IIB	Negative	9	Positive	Negative	16,18	RH + LND	CCRT	Yes	8	Yes, 2mo
15	CX16	50	cervix	SCC	IIA	Negative	6.5	Negative	Negative	16	RH + LND	RT	No	20	Yes, 12mo
16	CX17	52	cervix	SCC	IIB	Positive	7	Positive	Negative	45	RH + LND	CCRT	No	21	Yes, 5mo
17	CX19	59	cervix	SCC	IB2	Negative	4.4	Negative	Negative	18	RH + LND	RT	No	13	Yes, 8mo
18	CX20	32	cervix	SCC	IIA1	Positive	1.8	Positive	Negative	16	RH + LND	CCRT	No	12	No
19	CX21	53	cervix	SCC	IB2	Negative	5.4	Negative	Negative	31	RH + LND	RT	No	12	Yes, 4mo
20	CX22	51	cervix	SCC	IB1	Negative	4.3	Negative	Negative	16	RH + LND	RT	No	10	No
21	CX24	27	cervix	AC	IB2	Positive	3.5	Positive	Negative	16	RH + LND	CCRT	Yes	17	Yes, 5mo

AC, adenocarcinoma; CCRT, concurrent chemo-radiation therapy; CT, chemotherapy; DFS, disease free survival; LND, lymph node dissection; RH, radical hysterectomy; RT, radiation therapy; SCC, squamous cell carcinoma; Vaginal RM, vaginal resection margin

* NA: not assessed

### Histogenetic characteristics and similarities between patient's and PDX tissues

**Figure 1:**
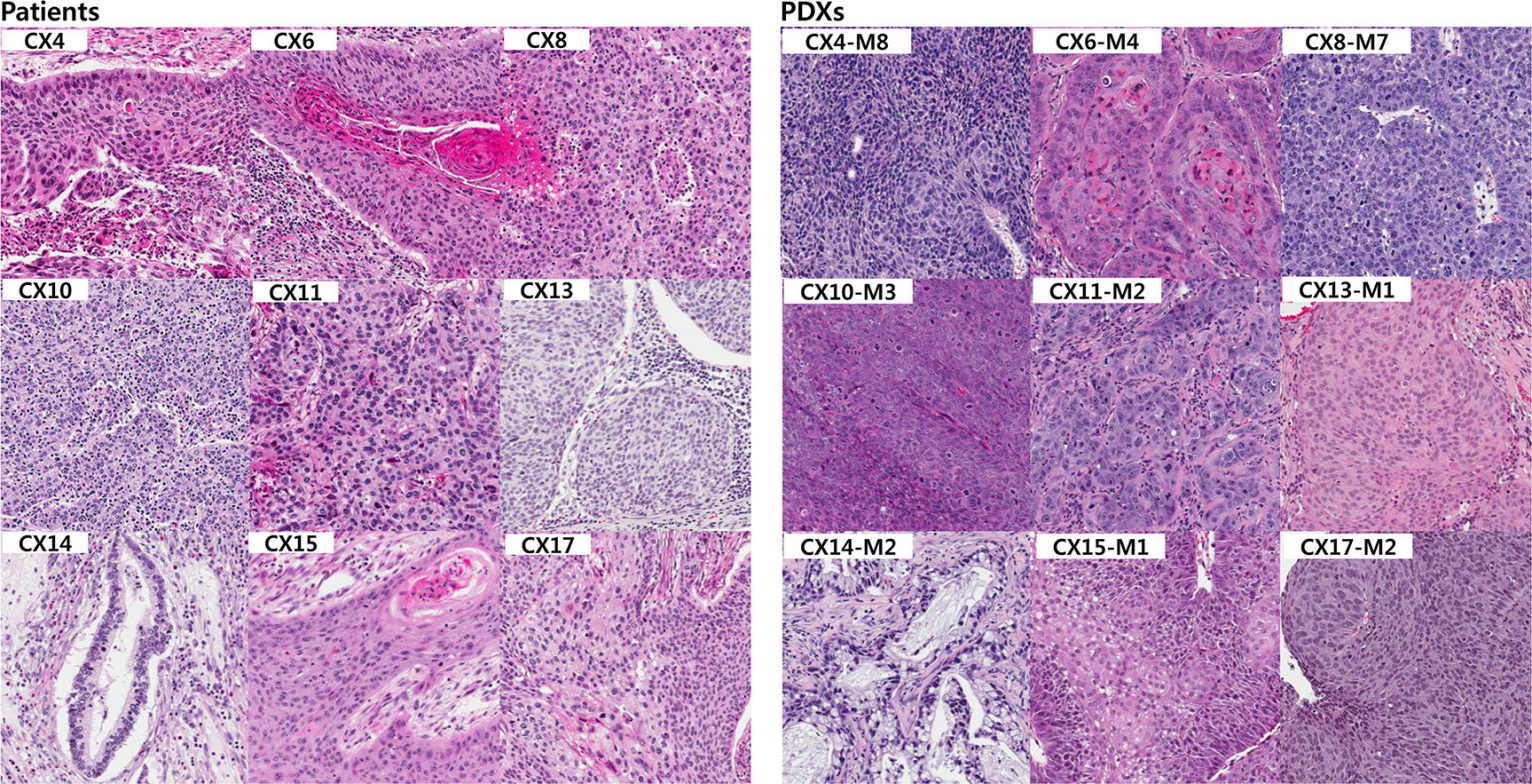
Histologic comparison between the patients and their PDX tumors Histologic analysis of the patient tumor samples (left panels) revealed eight were squamous cell carcinoma including both keratinizing and non-keratinizing types. The CX14 tumor sample was histologically endocervical adenocarcinoma (H&E, ×200). There was little difference between the histologic findings of the PDX tumor samples (right panels) and their corresponding patient samples.

Histologic evaluation of the engrafted tumors was performed in 9 PDXs after the mice were sacrificed. A blinded review of histology of tumors from patients and PDXs was done by three pathologists (S S., YL C. and S K.). Microscopic examination revealed the retained histological characteristics of the tumors - either squamous cell carcinoma or endocervical adenocarcinoma - despite the passage of lineage (Figure 1). Eight patient-PDX tumor pairs (CX4, 6, 8, 10, 11, 13, 15 and 17) showed histology of squamous cell carcinoma. Squamous cells with nuclear pleomorphism, intercellular bridges and relatively distinct cell borders were observed. Seven pairs were histologically graded as moderately differentiated squamous cell carcinoma, while one pair (CX11) were graded as poorly differentiated considering its profound nuclear pleomorphism and marked mitotic activity. Two pairs (CX6 and 15) were sub-classified as keratinizing type since they had abundant keratin pearls. Others were diagnosed as non-keratinizing type squamous cell carcinoma. There was a single pair showing histology of adenocarcinoma (CX14). Tumor cells architecturally forming relatively well-formed glands were observed in both patient and PDX tumors. However, a tendency to progress to poorly differentiated histologic characteristics was observed regardless of sample source as has been reported previously [5], suggesting that there is a clonal selection that divides actively to form a new tumor in the host nude mice toward more aggressive metastatic tumors. Tumor cells in PDX samples had more hyperchromatic nuclei with scanty cytoplasm. In CX14 PDX tumor (adenocarcinoma), tumor glands were more irregular in shape and situated back to back closely with little intervening stroma.

We subsequently investigated whether the genomic features of the original tumors were retained by their PDX counterparts by using array comparative genomic hybridization (aCGH) and short tandem repeat (STR) analysis. The aCGH profiles demonstrated that all PDX tumors faithfully conserved the genomic DNA alterations observed in the corresponding patient tumors (Supplementary Figure S1). The STR profiles of 7 PDX counterparts (i.e., CX4-M1, CX8-M1, CX10-M1, CX11-M1, CX13-M1, CX15-M1, and CX17-M1) were identical to their respective original human tumors at all STR loci (Supplementary Table S2). To determine whether the genomic statuses of the PDXs were accurate and without cross-contamination, we performed a quality control testing for the xenografts. The mean Ct values for the human *ALB* were much lower than those for the mouse *ALB* for all 9 PDXs, indicating human DNA was much more abundant than mouse DNA in the PDX tumor tissues (data not shown).

### Identification of aberrant *HER2* amplification in one case of PDX model

**Figure 2:**
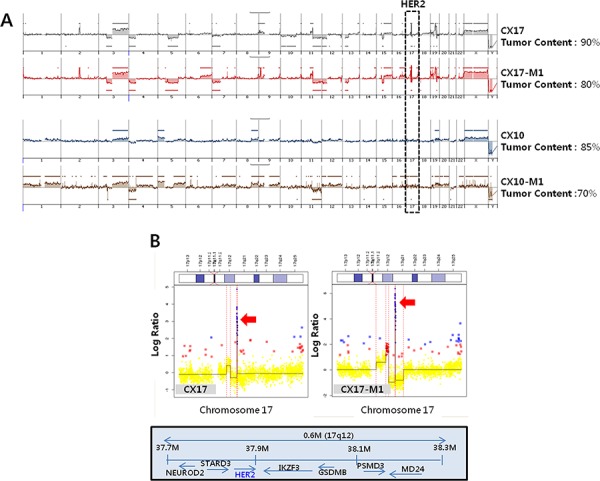
*HER2* amplification in a PDX (CX17) **A.** The recurrence of copy number alteration is plotted on the y-axis, and each probe is aligned along the x-axis in chromosomal order. Amplification of gene copy numbers are depicted in the black box (*HER2*). Note the similarity between the genomic profiles of the original tumors and their PDX counterparts. **B.** Details of the array comparative genomic hybridization profile of the chromosome 17 amplicon containing the amplified *HER2* oncogene comparing the original tumors and their PDX counterparts. Red dots represent gain and blue dots represent amplification for each probe aligned along the chromosome. The red arrows indicate *HER2* amplification in chromosome 17.

We analyzed single nucleotide variations, copy number variation, and translocation with genomic DNA in all 9 cervical cancer patient samples using Cancer Panel, a targeted next-generation sequencing-based assay, which assessed all exons from 81 cancer-related genes and 31 introns from 5 genes recurrently rearranged in cancer. However, no significant targetable oncogenic genetic alterations were detected in our samples when we analyzed genes reported to be mutated in the COSMIC database (http://cancer.sanger.ac.uk/cancergenome/projects/cosmic) (Supplementary Table S3). Interestingly, the Cancer Panel revealed a single case with *HER2* amplification (Supplementary Table S3). We investigated whether the genetic features of the original tumors were retained by their CX17 PDX counterparts by using aCGH. Both PDXs and the original tumors exhibited conserved aCGH profiles including the *HER2* amplicon localized on chromosome 17q21-22 in comparison to the other cases (Figure 2A and 2B).

### Incidence of HER2 expression in human cervical cancers

**Figure 3:**
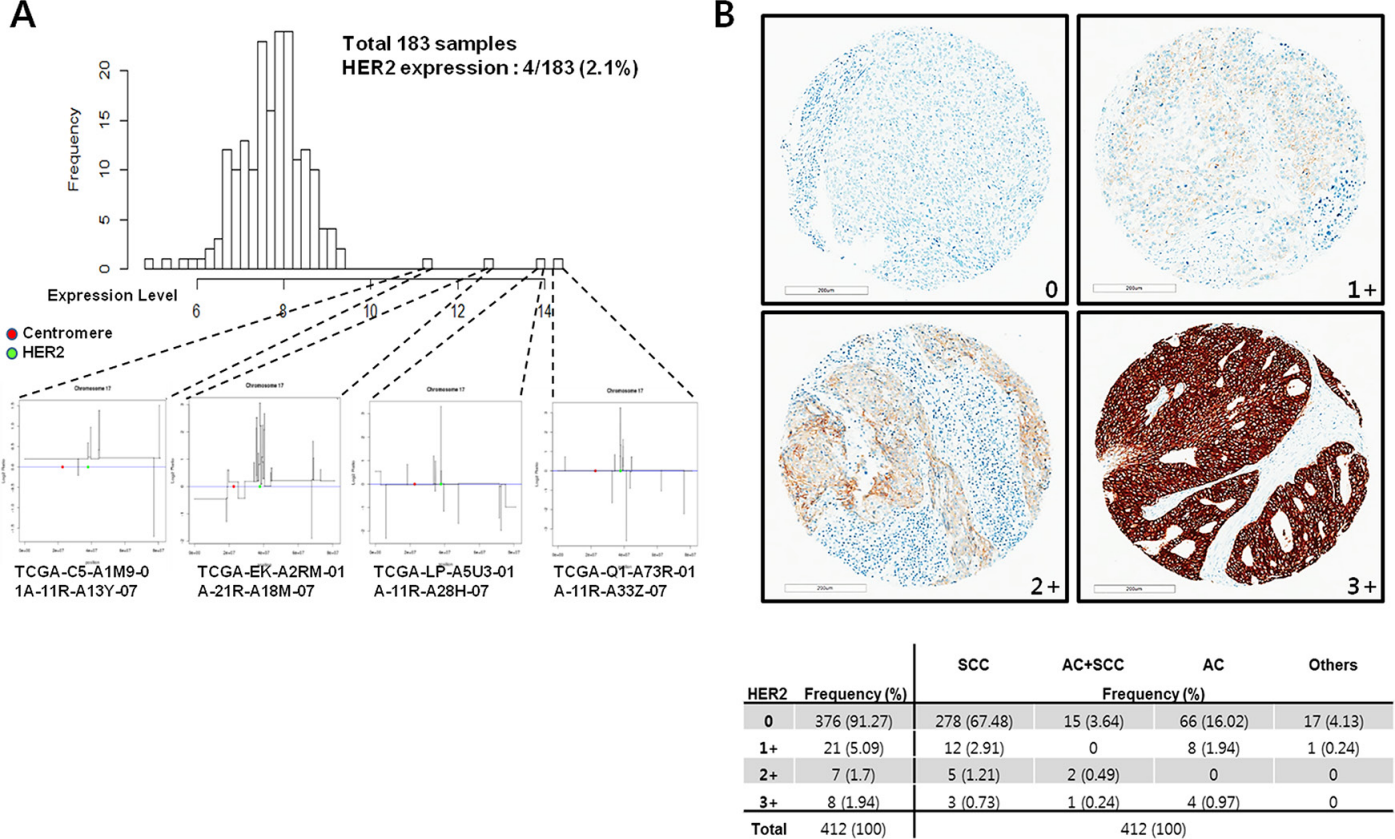
Incidence of HER2 expression in human cervical cancers **A.** In order to better characterize the HER2, we analyzed a large (*N* = 183) public microarray with the gene expression profiles (i.e., the Cancer Genomic Atlas) of cervical cancer patients. We investigated 4 cases with the highest HER2 expression levels that harbored *HER2* amplification. Red and green dots indicate the centromere and *HER2*, respectively. **B.** Frequency of HER2 expression in 412 cervical cancer patients (lower table). TMA core displaying membranous HER2 staining (From 0 to 3+) in 412 cervical cancer patients. score 0, no membrane reactivity; score 1+, group of tumor cells with weak or incomplete membrane reactivity; score 2+, group of tumor cells with weak to moderate membrane reactivity; score 3+, group of tumor cells with strong membrane reactivity. Scale bar = 200 μm.

To check the real proportion of HER2 expression in cervical cancer, we analyzed the Cancer Genomic Atlas database. We found that amplification and expression of HER2 was detected in 4 of the 183 available samples (2.1%; Figure 3A). To confirm the protein expression in human cervical cancer, we performed immunohistochemistry in tissue microarray (TMA) composed with 412 human cervical cancer tissues. Among the 412 cases analyzed, 15 cases (3.6%) were scored 2+ and 3+ (Figure 3B). HER2 was predominantly expressed in the cell membrane and representative results of immunohistochemical staining are shown in Figure 3B.

To determine whether *HER2* was amplified in other available cervical cancer cell lines (i.e., HeLa, SiHa, ME-180, MS751, and Caski), we analyzed the *HER2* copy number by quantitative polymerase chain reaction (qPCR). Only the CX17 patient's tumor but not the cervical cancer cell lines exhibited a high *HER2* copy number (Supplementary Figure S2). This suggests that only this PDX model can be used to investigate HER2-targeted therapeutics.

### Analysis of a case of *HER2*-amplified PDX

**Figure 4:**
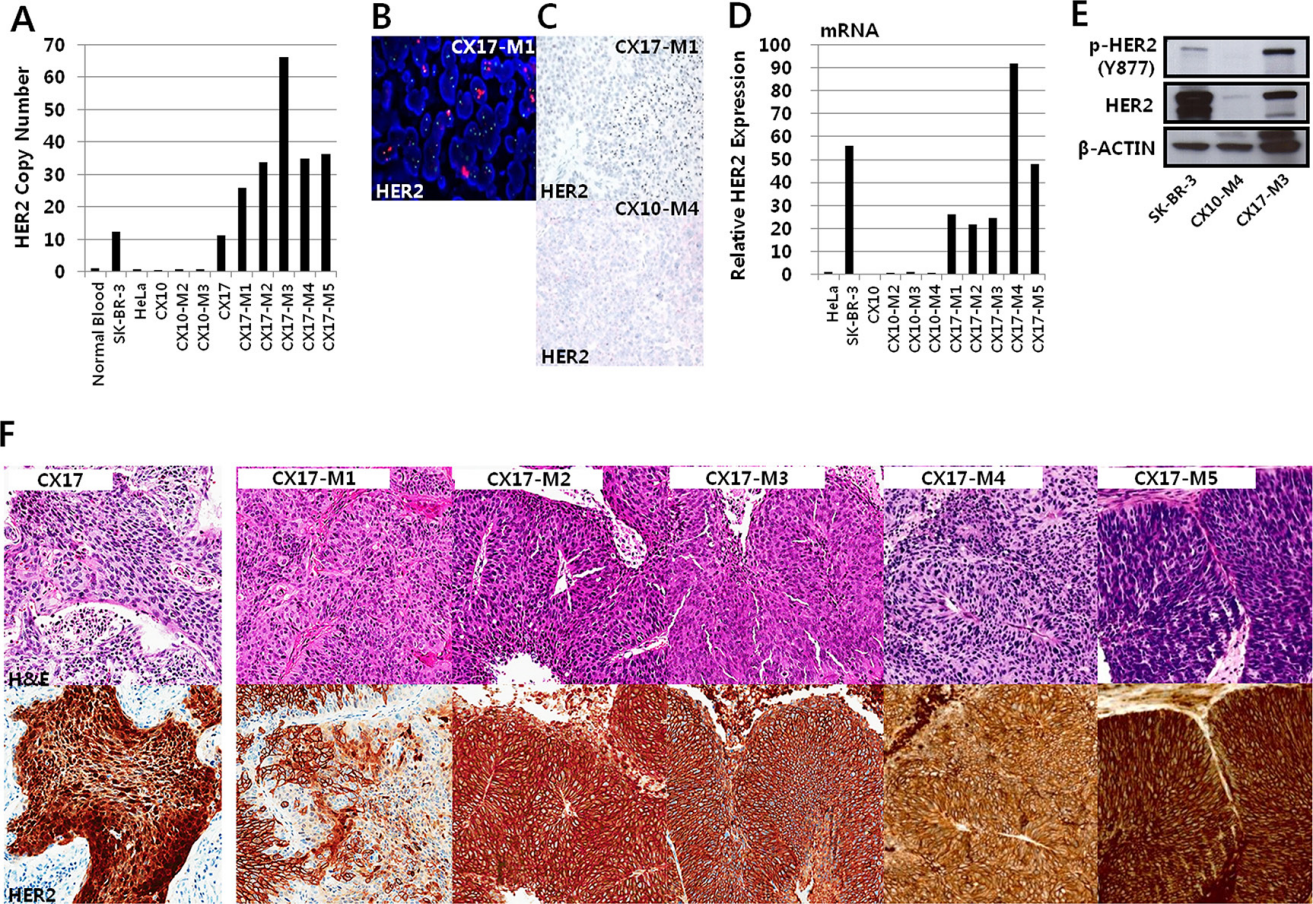
HER2 expression of CX17 PDX originating from the CX17 patient **A.** Quantitative polymerase chain reaction (qPCR) gene copy number analysis to detect *HER2* amplification. A breast cancer cell lines (SK-BR-3) was used as positive control to HER2 expression. **B.** Fluorescence *in situ* hybridization of *HER2* revealed amplification in many tumor cells (red dots). **C.** Silver *in situ* hybridization of *HER2* shows amplification of *HER2* in CX17-M1 (black dots in upper panel). **D.** Quantitative reverse transcription PCR to identify HER2 expression in CX10, CX10 PDXs, and CX17 PDXs. **E.** HER2, phosphorylated HER2, and β-actin as a loading control were analyzed by western blotting. **F.** The tumor histology did not show any significant difference with serial passages in the HER2-staining areas (hematoxylin and eosin, ×200, upper panels). Immunohistochemistry for HER2 revealed the transition of expression from nuclear and cytoplasmic to membranous expression with serial passages (lower panels). (HER2, ×200).

In the CX17 sample which exhibited *HER2* amplification in the genomic study, the tumor was histologically defined as a stage IIB squamous cell carcinoma. The patient was a 52-year-old woman with a 7-cm mass in her cervix. She received radical hysterectomy followed by concurrent chemo-radiation therapy, and no recurrence was detected until the 21 month postoperative follow-up. qPCR showed that *HER2* amplification was detected in CX17 case but not in CX10 (Figure 4A). *HER2* amplification in CX17-M1 was also demonstrated by fluorescence *in situ* hybridization (Figure 4B) and silver *in situ* hybridization (Figure 4C), but not in CX10 which has no HER2 expression.

To study the stability of the PDXs after serial transplantation, HER2 gene amplification and mRNA expression were analyzed in the serial passages and were compared with the expression profiles of the original tumors. HER2 gene amplification and mRNA expression remained very stable throughout sequential *in vivo* passages (Figure 4A and 4D). Moreover, HER2 expression was detected in CX17 PDX but not CX10 PDX according to western blotting and immunohistochemical staining results (Figure 4E and 4F). Although the tumors histologically showed few differences between the patient and serial passage samples on hematoxylin and eosin staining (Figure 4F, upper panel), the HER2 immunohistochemical staining pattern and intensity exhibited dramatic differences. The patient's tumor sample showed mainly cytoplasmic and nuclear staining for HER2 with small portions of membranous staining; 70% of the total tumor volume was stained (intensity: strong, 30%; weak, 40%; Figure 4F, left panel). However, in the first PDX tumor (CX17-M1), 70% of the tumor cells showed positive membranous HER2 immunostaining (strong intensity) and the remaining 30% showed weak or moderate cytoplasmic staining. Furthermore, all tumor cells from 4 subsequent PDX passages (CX17-M2-M5) showed strong HER2 membranous positivity (>95%; Figure 4F, lower panel), suggesting the selection of *HER2*-amplified clones had occurred with serial passages. HER2 immunohistochemical staining of the non-amplified case (CX10) showed weak cytoplasmic staining in both the patient sample and PDXs (Supplementary Figure S3). We also evaluated HER2 immunohistochemical positivity in 22 metastatic cervical cancer samples from patients (Supplementary Table S4). Among 22 metastatic samples, a single case that metastasized to the rectal wall 1 year after hysterectomy and subsequently to the colon exhibited HER2 positivity on immunohistochemical staining; 20% of the tumor cells in both metastatic samples were positive, showing a membranous staining pattern (Supplementary Figure S4). These results support the possibility of the clinical application of HER2 inhibitors for cervical cancer treatment.

### Efficacy of anti-EGFR therapy in a *HER2*-amplified PDX

**Figure 5:**
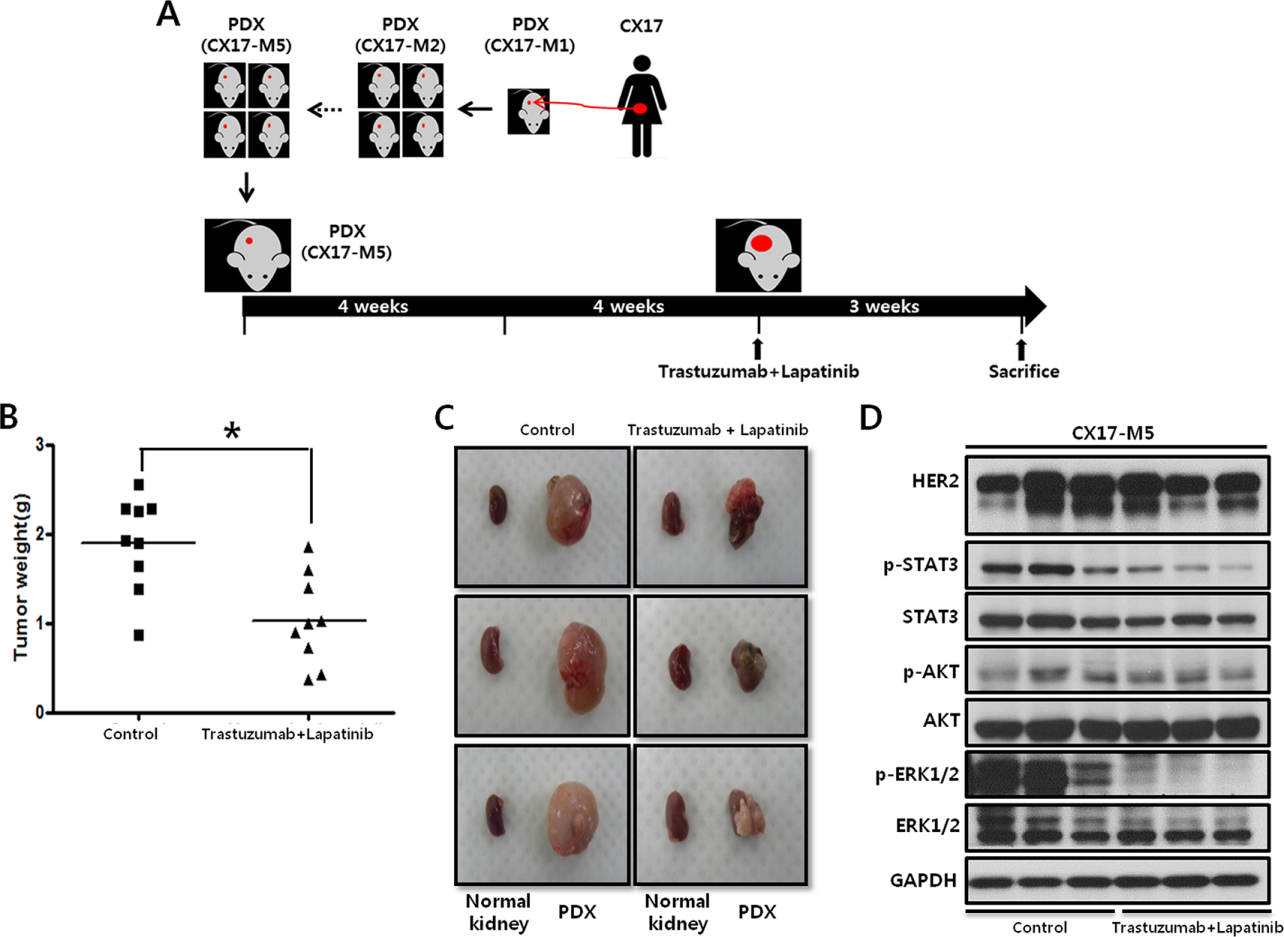
Effect of dual HER2 inhibitors administration on tumor growth in CX17 PDX **A.** Schematic that illustrates the CX17 PDX experimental design. **B.** Female BALB/c nude mice bearing the CX17 tumor tissue received the vehicle control (*N* = 10) or a combination of 10 mg/kg trastuzumab 2 days per week and 100 mg/kg lapatinib daily (*N* = 10) as indicated. *P = 0.013. **C.** Images of tumors from mice treated with the vehicle control or a combination of trastuzumab and lapatinib. In each picture, the small left piece is the normal kidney (i.e., no tumor transplanted), and the large right one is the developed PDX. **D.** Western blot analysis results with the indicated antibodies of representative tumors tissues taken after sacrifice (CX17 PDXs).

To assess the validity of HER2 as a therapeutic target in *HER2*-amplified cervical cancer, we used the CX17 PDX to evaluate the efficacy of 2 HER2 inhibitors by analyzing the inhibition of tumor growth. Trastuzumab and lapatinib are US Federal Drug Administration-approved HER2-inhibitors widely used to treat *HER2*-amplified breast cancers [15]. Compared to the untreated tumors, the combination of trastuzumab and lapatinib significantly reduced tumor weight (−50%; *P = 0.013; Figure 5A–5C). Moreover, trastuzumab and lapatinib combination decreased the phosphorylation of mitogen-activated protein kinase (Phospho-p42/44 MAPK [Thr202/Tyr204]) and STAT3 (Phospho-Stat3 [Tyr705]), but not AKT (Phospho-AKT [Ser473]) in the CX17 PDX (Figure 5D) [16].

## DISCUSSION

In this study, we established histopathologically and genomically homologous PDX models for human cervical cancer and found a single case with aberrant HER2 amplification and expression. Subsequent histopathologic and genomic characterization of the tumors in addition to monitoring response to anti-HER2 therapy in PDXs was performed. These results strongly suggest that the dual administration of a HER2 small-molecule inhibitor and a monoclonal antibody directed against the HER2/neu receptor is beneficial for the treatment of *HER2*-amplified cervical cancer in clinical settings.

Orthotopic tumor implantation may also confer a translational advantage, as the tumor develops in the same anatomic microenvironment [17]. However, the generation of orthoxenografts is more labor intensive and expensive, requires complex surgery, and often requires imaging methods to monitor tumor growth [18]. Meanwhile, tumor implantation in the subrenal capsule yields an impressive engraftment success rate and requires a simple surgery, which is one of the most important variables for studies seeking to implement real-time PDX data for personalized cancer treatment [5]. Although a heterotopic cervical cancer PDX models are useful for preclinical evaluation of personalized medicine, they are still limitations in translational cancer research. Heterotopic sites in the subcutaneous flank, mammary fat pad, and sub-renal capsule may lack the same microenvironment seen in the orthotopic sites within the ovary or peritoneum [19]. However, we first established cervical cancer PDX model with the higher subrenal capsule engraftment rate, low cost, recapitulating patient clinicopathologic features, tumor histology, and genomic characterization for at least 8 passages. To date, cervical cancer PDXs using subrenal implantation have not so studied despite their potential benefits for drug testing and treatment response monitoring. *Hiroshima et al*. [10] identified that the orthotopic model of cervical cancer, but not subcutaneous implantation mimics the patient metastatic pattern. The point is whether our cervical cancer PDX models reflect the patient metastatic pattern. Many papers identified that subrenal implantation model proved to be a suitable model to follow the metastatic process in prostate, renal, and colon cancer [20–22], suggesting that our cervical cancer PDX models would be suitable to mimic not only *in vivo* tumorigenicity, but also the metastatic pattern than other heterotopic PDX model. Thus, the present results support the value of cervical cancer PDXs using subrenal implantation for preclinically predicting drug responses.

In contrast to breast cancer, the prognostic value of HER2 in cervical cancer remains controversial. HER2 was associated with poor prognosis in 126 cervical cancer patients with stage IB/IIA disease and good prognosis in 55 cervical cancer patients with stages I–IVA disease [4]. *HER2* was amplified in a small percentage (2.1%) of genetically unselected cervical cancers from the Cancer Genomic Atlas database and its protein overexpression was 3.6% of cervical cancer patients in tissue microarray samples (Figure 3). These results are supported by work recently published by Ojesina *et al*. [12], who observed *HER2* amplification in a small percentage of cervical cancer patients and evidence of *HER2* activation by somatic mutation, amplification, and human papilloma virus (HPV) integration in cervical cancer. One of our patient samples exhibited *HER2* amplification, which was progressively enriched with serial passages. Although absolute numbers remain small, it is evident that some cases with *HER2* amplification either in cervical cancer PDXs or in patients will not respond to chemo-radiation. Another interesting result is the transition of nuclear and cytoplasmic HER2 expression to membranous expression with serial passages of the tumors in cervical cancer PDXs. Some reports indicate HER2-negative cervical cancers transform into HER2-expressing ones when they recur or metastasize [13, 23]; this transition of the HER2 tumor immunoprofile could be a therapeutic target. In one case report, a HER2-negative cervical cancer exhibited HER2 positivity after peritoneal metastasis; the patient had undergone combined trastuzumab and lapatinib treatment, and follow-up imaging revealed a dramatic treatment response [11]. There are two possible explanations for the transition of HER2 expression. First, a minor tumor clone originally expressing HER2 has a selective advantage with serial passages. One study indicates HER2-positive cells may have a selective advantage over HER2-negative cells both *in vitro* and *in vivo* [24]. Second, tumor cells previously not expressing HER2 began expressing HER2 after serial passaging. Some colon cancer cases exhibit cytoplasmic HER2 overexpression as was found in a patient tumor sample (CX17) in this study [25]. The accumulated *HER2* protein in the cytoplasm can migrate to the membrane under the influence of the unfolded protein response [26]. In breast cancer, 6% of HER2-negative primary tumors convert to HER2-expressing tumors during metastasis [27]. However, as our molecular data show increases in HER2 DNA, mRNA, and protein, it can be concluded that a *HER2*-amplified clone proliferated with serial passaging in the PDXs.

To date, we can find only one clinical study in cervical cancer patients to evaluate the therapeutic effects of anti-HER2/neu therapy. Monk *et al*. reported that only pazopanib alone improved progression-free survival, whereas the combination of lapatinib and pazopanib was not effective [28]. However, *HER2*-positive and *HER2*-negative patient groups were not classified, and HER2 inhibitors were administered to all advanced-stage cervical cancer patients. It is doubtful any patients with *HER2*-amplified cervical cancer had the potential to benefit from HER2-targeted therapy. In this study, we used a dual therapy with trastuzumab and lapatinib instead of anti-HER2 monotherapy. Previously, we found that no significant inhibition of CX17 PDX tumor growth was observed following treatment with trastuzumab or lapatinib alone compared to the vehicle-treated controls (data not shown). Moreover, treatment with trastuzumab alone down-regulated HER2 receptor expression (Supplementary Figure S5), suggesting trastuzumab-mediated HER2 receptor degradation may be the key to resistance acquisition, which inevitably occurs in *HER2*-amplified cervical cancer. The results of Scaltriti [16] and this study show that trastuzumab can down-regulate HER2 receptor, whereas the tyrosine kinase inhibitor, lapatinib, induces HER2 accumulation at the cell surface (Supplementary Figure S5). These results confirm trastuzumab alone may be not as effective as trastuzumab and lapatinib combination therapy in *HER2*-amplified cervical cancer. Moreover, Lapatinib in combination with trastuzumab to HER2-overexpressing breast cancer cells SKBR3 and MCF7-HER2, inhibited mitogen-activated protein kinase (MAPK) phosphorylation and *in vitro* and *in vivo* tumor growth [16], suggesting that a mechanism of action of the combination may be clinically relevant and exploitable in the therapy of patients with HER2-positive tumors. Although we checked the effect of 2 HER2 inhibitors on tumor growth *in vivo* in HER2-overexpressed cervical cancer PDX models, since our cervical cancer PDX is treatment naïve, we suggest that cervical cancer PDXs would respond to cisplatin-based chemotherapy or radiation therapy. Assuming cervical cancer PDXs accurately mimic the human situation, the tumor regression produced by trastuzumab and lapatinib combination therapy demonstrates the potential benefit of this therapeutic regimen if evaluated in clinical trials.

Currently, there are no clinical trials registered to examine dual anti-HER2 therapies in *HER2*-amplified cervical cancer. The preclinical data reported herein suggest combination therapy with trastuzumab and lapatinib induces significant *in vivo* anti-tumor activity and overcomes the potential for cervical cancer to harbor trastuzumab resistance induced by treatment with trastuzumab alone. Furthermore, our preclinical findings in *HER2*-amplified cervical cancer PDX models may accurately predict the outcomes and treatment response of cervical cancer patients.

## MATERIALS AND METHODS

### Patient tissue samples

The tumor samples from patients with cervical cancer were taken from fresh surgical specimens immediately after radical hysterectomy and lymph node dissection. All patients provided informed consent, and the study was conducted with institutional review board approval (IRB File No. 2009-09002). The patients' clinical information was obtained from medical records, including age, menopausal status, history of neoadjuvant or adjuvant therapy, and disease-free survival. The patients were followed up every 3 months, and examinations included a Pap smear and blood test for tumor markers (TA-4) in addition to imaging studies including magnetic resonance imaging and positron emission tomography to detect disease progression.

### Cell lines

The HeLa, SiHa, ME-180, MS751, Caski, and SK-BR-3 (*HER2*-amplified breast cancer) cell lines were obtained from the American Type Culture Collection (Rockville, MD, USA) and maintained in Dulbecco's modified Eagle medium supplemented with 10% fetal bovine serum and Dulbecco's modified Eagle's medium/Ham F12 1:1 (DMEM/F12) supplemented with 10% fetal bovine serum at 37°C in 5% CO_2_. All of the cell lines used were authenticated via short tandem repeat (STR) profiling before beginning a new series of experiments, and kept in culture for <3 months.

### Establishment of PDXs

Female BALB/c nude mice were purchased from ORIENT BIO (Sungnam, Korea). The tumor samples were collected after surgery and cut into 1-mm^3^ pieces in phosphate-buffered saline. Subrenal capsule xenografts were made from the human cervical tumor tissues grafted underneath the renal capsules of female BALB/c nude mice (*N* = 5 per tumor sample) as described previously [29]. When the tumors appeared about 2 cm at the graft site or mice became moribund after grafting, the mice were euthanized and the tumors were collected; these samples were considered tumorigenic and designated “M1”. Tumorigenic samples were serially passaged *in vivo* to make “M2” PDX tumors. Tumor tissue samples were stored in liquid nitrogen. This study was approved by the Institutional Animal Care and Use Committee of the Samsung Biomedical Research Institute, which is an accredited facility of the Association for Assessment and Accreditation of Laboratory Animal Care International (protocol No. H-A9-003) and abides by the Institute of Laboratory Animal Resources Guide.

### HPV genotyping

A polymerase chain reaction (PCR)-based HPV DNA microarray was performed for all 21 patients' tumor samples in formalin-fixed paraffin-embedded blocks by using a DNA chip (Biometrix Technology Inc., Chuncheon, South Korea) according to the manufacturer's instructions; 15 high-risk HPV types (HPV 16, 18, 31, 33, 35, 39, 45, 51, 52, 53, 56, 58, 59, 66, and 68) and 9 low-risk HPV types (HPV 6, 11, 34, 40, 42, 43, 44, 54, and 70) were detected [30].

### Histologic examination and immunohistochemistry

Hematoxylin and eosin (H&E) staining was performed on all paraffin blocks from the tissue samples obtained both from patients and PDXs. The PATHWAY^®^ anti-HER2/neu (4B5; Roche, Basel, Switzerland) antibody was used for HER2 immunohistochemical staining. Tissue sections (3 mm) were deparaffinized and rehydrated, and antigen retrieval was performed for 40 min in citrate buffer (pH 6.1) at 95°C. Diaminobenzidine was used as the chromogen, and the sections were counterstained with hematoxylin. The BenchMark XT automated slide processing system (Ventana Medical Systems, Tucson, AZ, USA) was utilized. HER2-expressing breast cancer tissues were used as a positive control.

### Array comparative genomic hybridization

Array comparative genomic hybridization was used to detect genetic variation in the form of deletions and duplications. An Agilent human whole-genome comparative genomic hybridization 8 × 60 K microarray platform from Agilent Technologies (Santa Clara, CA, USA) was utilized. Tumor and control DNA were labeled with Cy5-dCTP and Cy3-dCTP, respectively, followed by DNA hybridization to a microarray. All slides were scanned on an Agilent DNA microarray scanner. Data were extracted from scanned images with Agilent CGH Analytic Version 6.5 software using the ADM-2 statistical algorithms with 6.0 sensitivity thresholds. Signal intensities were normalized by comparing the tumor samples with normal male genomic DNA (Promega, G147A) and subsequently analyzed as described previously [9].

### Short tandem repeat analysis

For STR genotyping, target DNA was amplified by multiplex PCR for 16 loci using the AmpFlSTR Identifier PCR Amplification Kit (Applied Biosystems, Foster City, CA, USA) according to the manufacturer's instructions. PCR products mixed with an internal size standard (GS-500 LIZ; Applied Biosystems) were electrophoresed on an ABI 3130xL Genetic Analyzer (Applied Biosystems) and analyzed with GeneMapper 4.0 software using the supplied allelic ladders (Applied Biosystems).

### Mutation analysis using the cancer panel

The Cancer Panel is a targeted next-generation sequencing assay that was developed, validated, and provided by the Samsung Genome Institute (Samsung Medical Center, Seoul, Korea); it includes all exons from 81 cancer-related genes and 31 introns from 5 genes recurrently rearranged in cancer. Using the Illumina HiSeq 2500 instrument, the captured libraries underwent paired-end high-depth sequencing (target > 800 × coverage). Data were analyzed using an automated bioinformatic pipeline designed to detect various genetic alterations including single nucleotide variations, insertion and deletion, gene amplification and deletion, and gene fusions.

### Quantitative real-time and quantitative reverse-transcription polymerase chain reaction

*HER2* copy number amplification was performed with a PRISM 7900HT Fast Realtime PCR system (Applied Biosystems). The primer sequences are provided in supplementary Table S1. All reactions were performed in triplicate. The PCR program was as follows: preheating at 50°C for 2 min; 95°C for 10 min; 40 cycles at 95°C for 15 s and 60°C for 1 min; and 1 cycle for melting curve analysis. RNA isolation and cDNA synthesis were performed using the RNeasy Mini Kit (Qiagen, Valencia, CA, USA) and SuperScriptIII first-strand kit (Invitrogen, Waltham, MA, USA) according to the manufacturers' instructions. The HeLa cell line, which is a HER2-negative cervical cancer cell line, was used as a calibrator for the relative quantification of HER2 expression level. The calculation equation and PCR protocol were the same as those used for copy number analysis.

### HER2 *in situ* hybridization

Silver *in situ* hybridization (SISH) was performed according to the manufacturer's protocols. Sequential *in situ* hybridization procedures for *HER2* and *CEN17* signal detection were conducted with the INFORM *HER2* DNA, and *CEN17* probes (Ventana Medical Systems). The Ventana ultraView SISH Detection Kit on a Ventana BenchMark XT automated slide stainer was also utilized according to the manufacturer's instructions. Fluorescence *in situ* hybridization was performed using dual-color DNA-specific probes from PathVysion™ (LSI^®^ *HER2* Spectrum Orange™ and *CEP17* Spectrum Green™; Abbott, San Francisco, CA, USA).

### Tissue microarray construction

H&E-stained slides of cervical cancer were reviewed and the appropriate tumor area was marked. The corresponding paraffin block was retrieved and the marked areas were matched. The cores of tumor areas were manually punched using a precision instrument (Labro TMA kit) and embedded into the recipient block with 60 microholes (diameter, 2 mm; depth, 5 mm). The microarray blocks were heated at 60°C for 30 min.

### Western blotting

Immunoblotting was performed using antibodies against the following: HER2 (Cell Signaling Technology, 2165), p42/44 MAPK (Cell Signaling Technology, 9102), Phospho-p42/44 MAPK (Thr202/Tyr204; Cell Signaling Technology, 4370), AKT (Cell Signaling Technology, 9272), Phospho-AKT (Ser473; Cell Signaling Technology, 9271), STAT3 (Cell Signaling Technology, 4904), Phospho-STAT3 (Tyr705; Cell Signaling Technology, 9145), β-actin (Santa Cruz, sc-47778), GAPDH (Santa Cruz, sc-25778), and anti-HER2 (phosphor Y877; Abcam, ab108371).

### HER2-targeted therapy

Tumor tissue from the CX17 patient was obtained following surgery and implanted underneath the renal capsules of female BALB/c nude mice as described above. After engraftment and tumor mass formation, the tumors were passaged and expanded by 5 generations (M5) with 2 cohorts each consisting of 10 mices. After implantation (8 weeks), the established tumors (*N* = 10 for each tumor sample) were treated with the following regimens: trastuzumab (Roche Genentech, San Francisco, CA, USA) 10 mg/kg twice weekly and lapatinib (LC Laboratories, Woburn, MA, USA) 100 mg/kg daily. After 3 weeks of treatment, the tumors grown under the subrenal capsule were harvested and weighed.

### Statistical analysis

The Mann–Whitney *U* test was used to evaluate the significances to compare differences among the groups for *in vivo* assay. All statistical tests were two-sided, and *P* values less than 0.05 were considered to be statistically. SPSS software (version 17.0; SPSS, Chicago, USA) was used for all statistical analyses.

## SUPPLEMENTARY FIGURES AND TABLES


