# Supplementary material for: HER2 as a novel therapeutic target for cervical cancer
Source: Oncotarget. 2015 Sep 21;6(34):36219–30. doi: 10.18632/oncotarget.5283 (PMC4742172; doi:10.18632/oncotarget.5283)
Supplement: Supplementary file 1 [file oncotarget-06-36219-s001.pdf]

SUPPLEMENTARY FIGURES AND TABLES

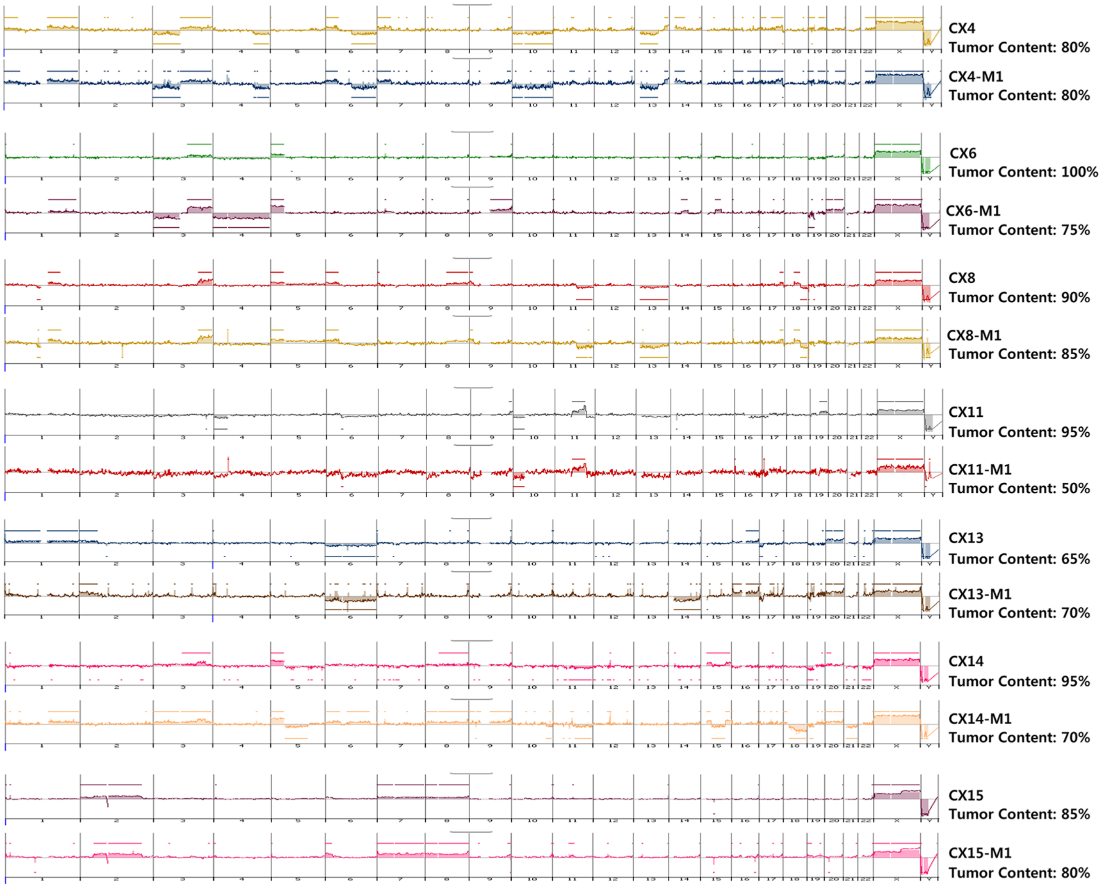

Supplementary Figure S1: Array comparative genomic hybridization results of 7 patient tumors and PDXs. Recurrence of copy number alteration is plotted on the y-axis, and each probe is aligned along the x-axis in chromosomal order. Note the similarity of genomic profiles between the original tumors and their PDX counterparts.

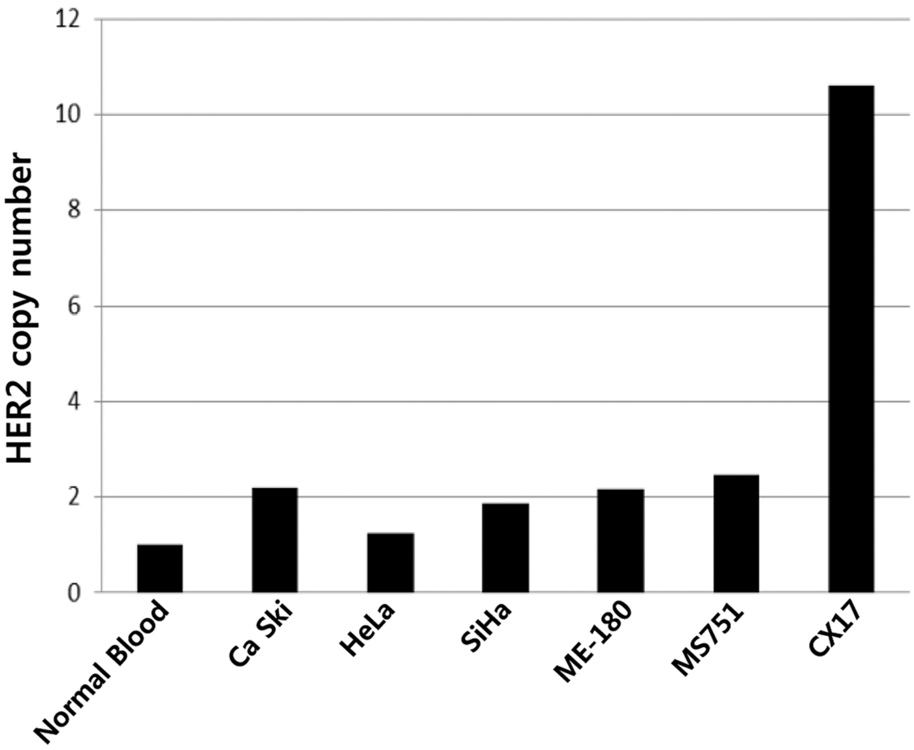

Supplementary Figure S2: Quantitative PCR gene copy number analysis in cervical cancer cell lines (HeLa, SiHa, ME-180, MS751, and Caski).

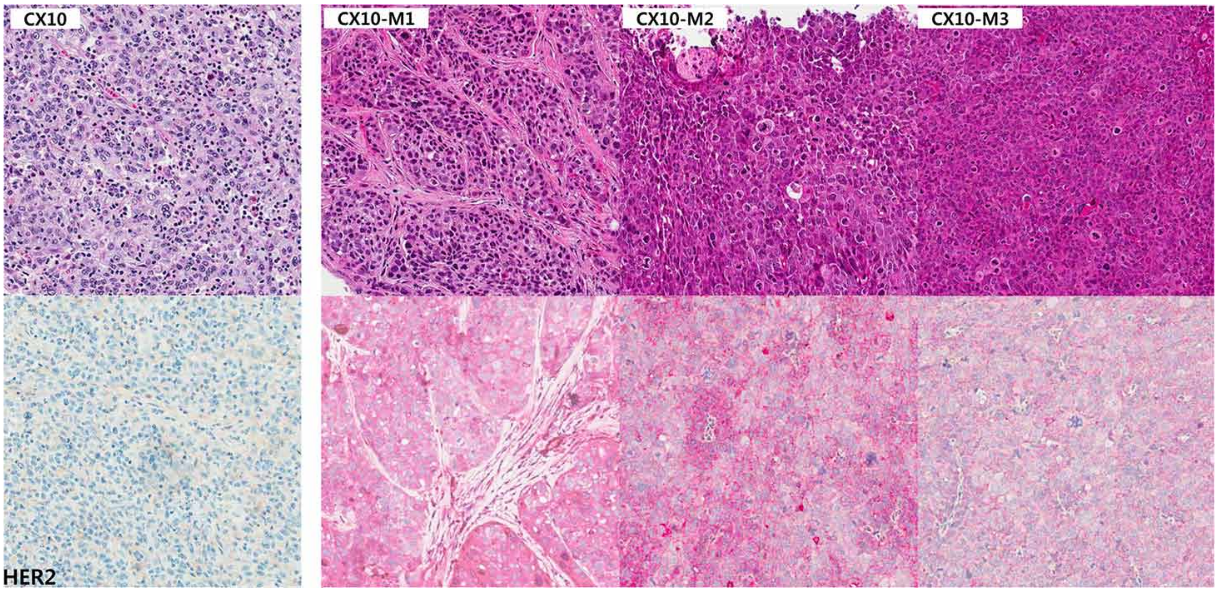

Supplementary Figure S3: Histology and HER2 immunohistochemistry of the *HER2*-non-amplified samples (CX10). HER2 immunohistochemical staining was negative in the patient tumor sample (CX10, lower panel). There was nonspecific weak staining with serial PDX passages (CX10-M1, CX10-M2, and CX10-M3, lower panels). The tumor histology following serial passaging shows little difference between them.

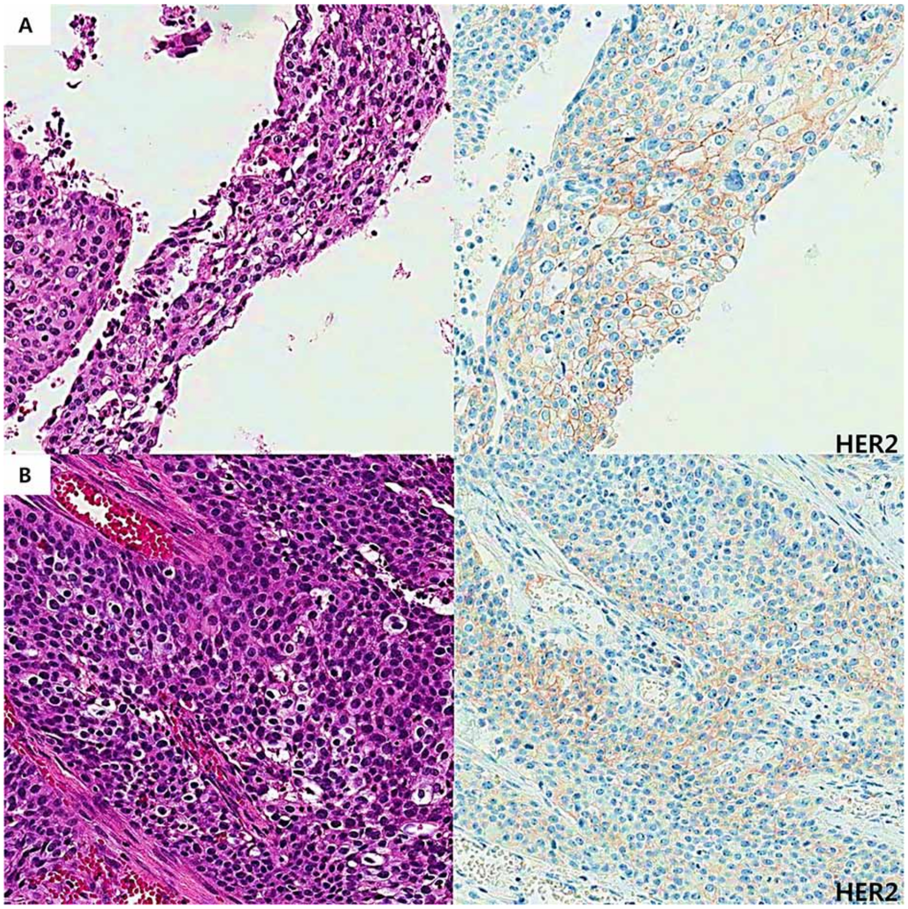

Supplementary Figure S4: Histology and HER2 immunohistochemistry of the rectal wall and colon metastasis samples of a cervical cancer patient. A. Immunohistochemical staining shows membranous HER2 positivity in the rectal wall squamous cell carcinoma metastasis from the cervix (HER2, ×200). **B.** Metastatic tumor cells in the colon show HER2 positivity (HER2, ×200).

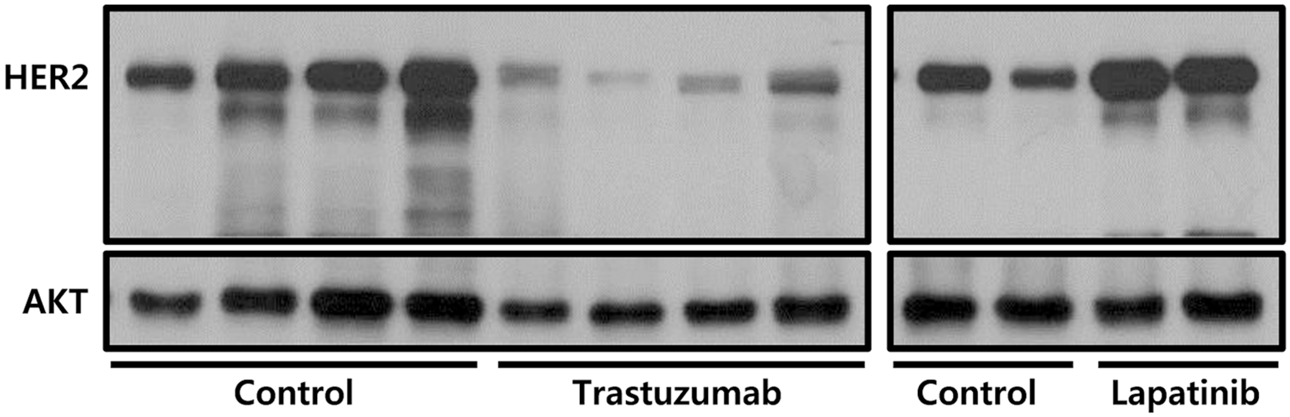

Supplementary Figure S5: Trastuzumab but not lapatinib induced HER2 degradation. Female BALB/c nude mice bearing the CX17 tumor tissue (CX17-M5) were treated with the control vehicle or trastuzumab 10 mg/kg twice weekly or lapatinib 100 mg/kg daily as indicated. Western blot analysis results with the indicated antibodies of representative tumors from CX17 PDXs at the end of treatment (8 weeks) are shown.

Supplementary Table S1: List of primer sequences used to generate qPCR/qRT-PCR expression data

***HER2* copy number (qPCR)**

Gene	Forward	Reverse
HER2	GGAGGATGTGCGGCTCG	CATGGTTGGGACTCTTGACCA
ALB (reference)	TGAAACATACGTTCCCAAAGAGTTT	CTCTCCTTCTCAGAAAGTGTGCATAT

HER2 expression (qRT-PCR)

Gene	Forward	Reverse
HER2	TGCCTGTCCCTACAACTACCTT	CCGCTGTGTTCCATCCTC
CTBP1(reference)	GGGAGATCCGCAGAGCCAT	GGTCCTTGTTGACACAGTTCTTC
UBQLN1 (reference)	AGTAAATCCTCAGCTACAGAATCCA	TTGCTTCACGGTTCAAAAATC

Supplementary Table S2: Short tandem repeat profiles of human patient tumors and PDXs

Locus	Chromosome Location	CX4	CX4-M1	CX6	CX6-M1	CX8	CX8-M1	CX10	CX10-M1	CX11	CX11-M1	CX13	CX13-M1	CX14	CX14-M1	CX15	CX15-M1	CX17	CX17-M1
D8S1179	8	10, 13	10, 13	10, 15	10, 15	15	15	11, 14	11, 14	11, 15	11, 15	13	13	13, 14	13, 14	11, 14	11, 14	10, 14	10, 14
D21S11	21q11.2-q21	30	30	30	30	30, 31.2	30, 31.2	28, 29	28, 29	31	31	30	30	28, 32.2	28, 32.2	30, 33.2	30, 33.2	29, 30	29, 30
D7S820	7q11.21-22	13	13	8, 14	8, 14	11, 12	11, 12	11	11	11	11	10, 11	10, 11	11	11	10	10	8, 10	8, 10
CSF1PO	5q33.3-34	10	10	10, 12	10, 12	11, 12	11, 12	12	12	10	10	11, 12	11, 12	12	12	11, 12	11, 12	9, 11	9, 11
D3S1358	3p	15	15	15	15	14, 17	14, 17	15, 16	15, 16	16, 17	16, 17	16	16	15, 16	15, 16	15, 16	15, 16	17	17
TH01	11p15.5	7, 8	7, 8	6, 9	6, 9	9, 9.3	9, 9.3	9	9	9	9	9	9	7	7	7, 9	7, 9	6, 7	6, 7
D13S317	13q22-31	11	11	8, 11	8	10, 11	10, 11	12	12	8	8	8, 14	8, 14	11, 13	11, 13	12	12	10, 11	10, 11
D16S539	16q24-qter	9	9	9, 11	9, 11	9	9	9, 13	9, 13	9, 11	9, 11	11, 12	11, 12	9, 10	9, 10	10, 11	10, 11	9, 11	9, 11
D2S1338	2q35-37.1	22, 23	22, 23	18	18	17, 25	17, 25	23, 25	23, 25	22	22	17, 19	17, 19	21, 25	21, 25	18, 20	18, 20	18, 24	18, 24
D19S433	19q12-13.1	15, 15.2	15, 15.2	12, 13.2	12, 13.2	13, 13.2	13, 13.2	13, 14.2	13, 14.2	13, 15.2	13, 15.2	13, 14.2	13, 14.2	13, 16.2	13, 16.2	14, 15.2	14, 15.2	12, 14	12, 14
vWA	12p12-pter	14	14	16, 17	16, 17	17	17	19	19	14	14	17, 18	17, 18	14, 18	14, 18	17, 18	17, 18	16	16
TPOX	2p23-2per	8, 11	8, 11	8	8	8	8	11	11	8, 9	8, 9	8, 11	8, 11	8, 11	8, 11	8, 9	8, 9	8, 9	8, 9
D18S51	18q21.3	14, 22	14, 22	12, 13	12, 13	14	14	15, 16	15, 16	21, 22	21, 22	17, 18	17, 18	13, 20	13	17	17	14, 18	14, 18
X	X:p22.1-22.3 Y:p11.2	X	X	X	X	X	X	X	X	X	X	X	X	X	X	X	X	X	X
D5S818	5q21-31	11, 13	11, 13	12, 13	12, 13	11, 13	11, 13	11	11	11, 13	11, 13	10, 11	10, 11	9, 12	9	10, 11	10, 11	9	9
FGA	4q28	24	24	23	23	17, 25	17, 25	23	23	21, 22	21, 22	21	21	22, 23	22, 23	21, 23	21, 23	23, 24	23, 24

Supplementary Table S3: Genomic profiles of cervical cancer patients

Sample	Chromosome	Position	Gene	Ref	Alt	Vaf	Variant
CX-4	chr3	37067297	MLH1	C	T	89.31	P403L
	chr4	153247366	FBXW7	C	T	68.69	R479Q
	chr3	178936091	PIK3CA	G	A	51.52	E545K
	chr4	1803564	FGFR3	C	T	46.41	R248C
	chr11	108188181	ATM	G	C	44.82	E2094Q
	chr2	30143236	ALK	G	A	42.17	P97L
CX-6	chr12	46123647	ARID2	C	A	48.97	P10T
	chr10	123256225	FGFR2	C	T	12.34	V563I
CX-8	chr17	29661936	NF1	G	T	28.54	D1965Y
CX-10	chr13	32914277	BRCA2	A	G	67.38	I1929V
	chr19	17950437	JAK3	G	C	9.77	I430M
	chr19	17952325	JAK3	G	C	9.61	L339V
CX-11	chr6	157528702	ARID1B	G	A	59.19	A2143T
	chr9	21971161	CDKN2A	T	C	45.19	H66R
	chr9	139413916	NOTCH1	G	A	26.47	R282C
CX-13	chr7	148525940	EZH2	C	G	29.78	E173Q
CX-14	chr19	1221213	STK11	TACA	T	58.8	YN246Y
	chr12	56482537	ERBB3	G	A	34.36	E332K
CX-15	chr9	139393636	NOTCH1	G	A	57.12	R2004C
	chr1	162746114	DDR2	G	A	47.71	R746Q
	chr15	90645555	IDH2	G	C	46.27	P23R
	chr9	21971161	CDKN2A	T	C	41.89	H66R
CX-17	chr1	11186787	MTOR	C	A	49.61	V2140L
	chr17	7579882	TP53	C	G	45.89	E11Q
	chr1	156846336	NTRK1	C	T	45.36	R593W
	chr13	32929042	BRCA2	C	G	7.89	A2351G
	chr4	153247157	FBXW7	C	T	7.64	splicing defect

Sample	Chr	Start	End	Gene	CNV	avg.L2R
CX4						
CX6						
CX8	chr19	1206888	1226679	STK11	del	–1.426
CX10						
CX11						
CX13	chr17	7569522	7579985	TP53	del	–1.804
CX14	chr17	7569522	7579985	TP53	del	–1.549
CX15						
CX17	chr17	37855737	37884329	ERBB2	amp	2.425
	chr19	40739406	40771239	AKT2	amp	1.543
	chr3	178916581	178952186	PIK3CA	amp	1.071
	chr4	1795633	1809435	FGFR3	del	–1.81

avg. L2R, average $\log_2$ R ratio; CNV, copy number variation.

Supplementary Table S4: Clinicopathological characteristics of 22 metastatic cervical cancer patients

No	Age	Metastasis site	Pathology	Pathologic stage	Surgery	Adjuvant therapy	Time to metastasis (months)	HER2 immunohisto-chemistry
1	62	Pubis	SCC	NA	ND	CCRT	12	Negative
2	44	colon	SCC	pT3cN0	RH+LND	CT	NA	Negative
3	31	brain	SCC	NA	ND	CT	16	Negative
4	59	lung	Small cell carcinoma, non-pulmonary type	NA	TAH	CT	96	Negative
5	64	colon	SCC	NA	ND	NA	*-	Negative
6	53	liver	SCC	pT1b1N0	RH+LND	CT	32	Negative
7	40	stomach	SCC	NA	ND	CCRT	10	Negative
8	51	liver	Lymphoepithelioma-like carcinoma	pT1b1N0	RH+LND	CT	36	Negative
9	73	small intestine	SCC	NA	ND	CCRT	*-	Negative
10	51	colon, ovary	SCC	ypT3cN0M1	RH+LND	CCRT	*-	Negative
11	31	Abdominal wall	SCC	pT2aN1	RH+LND	CCRT	6	Negative
12	67	rectum	AC	pT1b1N0	RH+LND	CCRT	19	Negative
13	50	rectum	SCC	pT1b2N0	RH+LND	CCRT	36	Negative
14	76	rectum	SCC	pT1b1N0	RH+LND	RT	33	Negative
15	62	lung	SCC	pT2bN0	RH+LND	RT	69	Negative
16	33	colon	SCC	pT1b2N0	RH+LND	CT	16	Negative
17	55	lymph node, brain	Mixed small cell neuroendocrine and squamous cell carcinoma	pT1b1N0	RH+LND	CCRT	28	Negative
18	46	lung	SCC	pT1b1N0	RH+LND	CT	37	Negative
19	37	chest wall	SCC	pT1b1N0	RH+LND	CCRT	52	Negative
20	47	liver	SCC	NA	ND	CT	60	Negative
21	42	Rectal wall, colon	SCC	pT1b1N0	RH+LND	CT	12	Positive
22	39	pelvic wall, lung	SCC	pT1b2N0	trachelectomy +LND	CCRT	6	Negative

AC, adenocarcinoma; CCRT, chemo-radiation therapy; CT, chemotherapy; LND, lymph node dissection; NA, not available; ND, not done; RH, radical hysterectomy; RT, radiation therapy; SCC, squamous cell carcinoma; TAH, total abdominal hysterectomy

*-, primary and metastatic tumors were diagnosed simultaneously.
